# Anorectal melanoma presenting as a polypoid lesion

**DOI:** 10.1055/a-2223-0584

**Published:** 2024-01-09

**Authors:** Qi Luo, Liansong Ye, Meiting Liang, Tingfa Peng, Bing Hu, Yi Mou

**Affiliations:** 1 34753 Department of Gastroenterology and Hepatology, Sichuan University West China Hospital, Chengdu, China; 2 Department of Gastroenterology, Armed Police Forces Hospital of Sichuan, LeShan, China

A 57-year-old woman presented with intermittent painless rectal bleeding for 1 year. Colonoscopy showed a polypoid lesion 2 cm from the anus ( Fig. 1 **a** ). Narrow-band imaging (NBI) revealed the presence of turbulent microvasculature in the surface of the lesion ( Fig. 1 **b** ). Enhanced computed tomography revealed that the rectum was of uneven thickness and showed local enhancement; there were no enlarged lymph nodes.

**Fig. 1:**
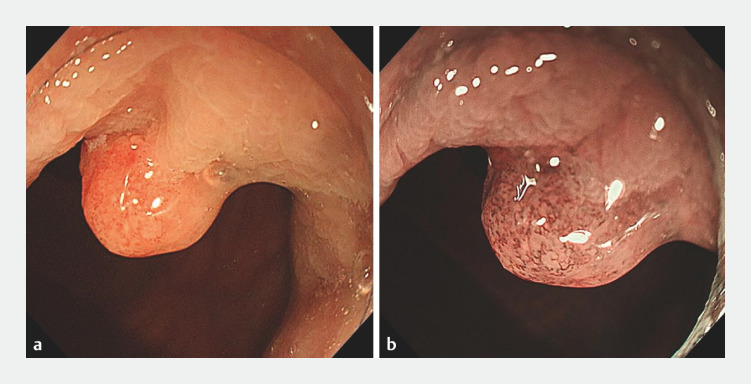
Colonoscopic images of a polypoid lesion 2 cm from the anus: **a** on white-light imaging; **b** on narrow-band imaging, showing the presence of turbulent microvasculature in the surface of the lesion.

In order to determine the nature of the lesion and resect it completely, endoscopic submucosal dissection was performed using a DualKnife ( Video 1 ). The procedure was successful, achieving en bloc resection of the lesion. Pathologic examination revealed a malignant intraepithelial proliferation of melanocytes ( Fig. 2 **a** ), and immunohistochemistry was positive for S100 ( Fig. 2 **b** ) and HMB45 ( Fig. 2 **c** ); the Ki-67 index was 60% ( Fig. 2 **d** ). The patient was therefore diagnosed as having a malignant melanoma.

Endoscopic submucosal dissection for an anorectal melanoma that presented as a polypoid lesion.Video 1

**Fig. 2:**
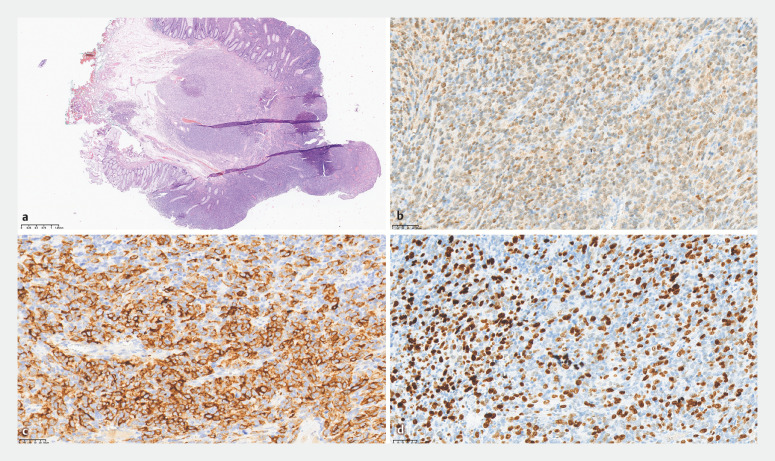
Histopathologic appearance of the resected lesion showing: **a** a malignant intraepithelial proliferation of melanocytes (hematoxylin and eosin [H&E] stained, ×20); **b,c** positivity on immunohistochemical staining for: **b** S100; **c** HMB45; **d** Ki-67 index of 60%.

Molecular testing revealed no *BRAF* , *NRAS* , or *KIT* gene mutations. Positron emission tomography–computed tomography showed no signs of tumor metastasis. Additional laparoscopic wide local excision was performed, yielding no residual tumor or lymph node metastasis. After 2 months, the patient received four cycles of adjuvant chemotherapy with temozolomide plus cisplatin. During 19 months of follow-up, she has remained well, and there has been no evidence of tumor recurrence or metastasis.

Anorectal melanoma (ARM) is an extremely rare malignancy, which accounts for only 0.5%–4.6% of all malignant anorectal neoplasms and 1.4% of all melanomas 1 2 . ARM is usually polypoid and often melanotic 3 4 . Amelanotic ARMs may be misdiagnosed as polyps or adenocarcinomas, which may contribute to their poor prognosis because of their highly aggressive potential. The overall median survival of ARM is reported to be 8–18.6 months 1 . This case highlights the importance of raising awareness of ARM, as having a high level of clinical suspicion could avoid misdiagnosis or delayed diagnosis, thereby improving prognosis.

Endoscopy_UCTN_Code_CCL_1AD_2AB

## Notes

Endoscopy E-Videos https://eref.thieme.de/e-videos
*E-Videos* is an open access online section of the journal *Endoscopy* , reporting on interesting cases and new techniques in gastroenterological endoscopy. All papers include a high-quality video and are published with a Creative Commons CC-BY license. Endoscopy E-Videos qualify for HINARI discounts and waivers and eligibility is automatically checked during the submission process. We grant 100% waivers to articles whose corresponding authors are based in Group A countries and 50% waivers to those who are based in Group B countries as classified by Research4Life (see: https://www.research4life.org/access/eligibility/ ). This section has its own submission website at https://mc.manuscriptcentral.com/e-videos .
